# Tracking of Tropical Intraseasonal Convective Anomalies: 1. Seasonality of the Tropical Intraseasonal Oscillations

**DOI:** 10.1029/2019JD030873

**Published:** 2020-02-03

**Authors:** Bohar Singh, James L. Kinter

**Affiliations:** ^1^ Colorado State University Fort Collins CO USA; ^2^ Center for Ocean‐Land‐Atmosphere Studies George Mason University Fairfax VA USA

**Keywords:** Multiple Object Tracking method, Madden‐Julian Oscillation, tropical intraseasonal oscillation, tracking of tropical intraseasonal oscillation, seasonality of tropical intraseasonal oscillation

## Abstract

A tracking algorithm based upon a multiple object tracking method is developed to identify, track, and classify Tropical Intraseasonal Oscillations (TISO) on the basis of their direction of propagation. Daily National Oceanic and Atmospheric Administration Outgoing Longwave Radiation anomalies from 1979–2017 are Lanczos band‐pass filtered for the intraseasonal time scale (20–100 days) and spatially averaged with nine neighboring points to get spatially smoothed anomalies over large spatial scales (~10^5^ km^2^). TISO events are tracked by using a two‐stage Kalman filter predictor‐corrector method. Two dominant components of the TISO (Eastward propagating and Northward propagating) are classified, and it is found that TISO remains active throughout the year. Eastward‐propagating TISO events occur from November to April with a phase speed of ~4 m/s and northward‐propagating TISO events occur from May to October with a phase speed of ~2.5 m/s in both the Indian and Pacific Ocean basins. Composites of the mean background states (wind; sea surface temperature, SST; and moisture) reveal that the co‐occurrence of warm SST and mean westerly zonal wind plays an important role in the direction of propagation and the geographical location of TISO events. In mean state sensitivity experiments with Sp‐CAM4, we have found that the seasonality of TISO in terms of the geographical location of occurrences and direction of propagation is primarily associated with the annual march of the maximum SST and low level zonal wind which tends to follow the SST.

## Key Points

- Multiple Object Tracking Method successfully detects and tracks TISO regardless of season and direction of propagation
- Co‐occurrence of warm SST and mean background westerly are important for the seasonality of TISO
- The seasonality of TISO primarily associated with the annual march of the maximum SST and low level zonal wind which tends to follow the SST

## 1 Introduction

The Tropical Intraseasonal Oscillation (TISO) refers to variability on the time scale of 20–100 days, intermediate between the time scales traditionally associated with weather and climate. TISO regulates the wet and dry spells of rainfall in the tropics (Carvalho et al., 2004; Jones et al., 2004; Lawrence & Webster, 2002; Sikka & Gadgil, 1980). TISO can be classified into two dominant components on the basis of seasonality: (a) the Madden‐Julian Oscillation (MJO, e.g., Madden & Julian, 1971, 1972) whose disturbances propagate eastward with average speed of ~5 m/s (Knutson & Weickmann, 1987; Weickmann et al., 1985) in all seasons but primarily in boreal winter (Jones et al., 2004; Wang & Rui, 1990; Yasunari, 1979) and (b) the Monsoon Intraseasonal Oscillation (MISO) or Boreal Summer Intraseasonal Oscillations (BSISO), whose disturbances propagate northward (Lau & Chan, 1986; Chen & Murakami, 1988; Gadgil & Srinivasan, 1990; Ferranti et al., 1999; Annamalai et al., 1999) during boreal summer with average speed ~1.3 m/s. The latter is also termed subseasonal variability of Indian summer monsoon rainfall (Krishnamurthy & Kinter, 2003; Raghavan, 1973; Ramamurthy, 1969).

The MJO (eastward TISO) exhibits considerable seasonality (Zhang & Dong, 2004). For example, eastward TISO events (MJO) occur primarily in the boreal winter season, when the solar zenith angle and the warmest sea surface temperature (SST) are south of the equator. MISO events are largely confined to boreal summer when the solar zenith angle and warmest SST are in the Northern Hemisphere. The seasonal migration of TISO is much stronger in the western Pacific Ocean as compared to the Indian Ocean. Low‐level mean moisture helps determine the phase speed and growth rate of the MJO. Comparisons of simulations from different AGCMs have suggested that simulated MJO‐like signals tend to be stronger in models whose mean seasonal cycles are stronger and whose mean precipitation is more realistically distributed with respect to SST (Slingo et al., 1996). The MJO appears to prefer low‐level and surface mean westerlies in both simulations (Inness et al., 2003). Zhang and Dong (2004) examined the relative role of SST and the large‐scale circulation in the seasonality of MJO (eastward propagating TISO). They were unable to identify any single mean background variable that could explain the seasonality of TISO in both the Indian and western Pacific Oceans. They found that the seasonality in MJO is best in the western Pacific Ocean, less apparent in the eastern Pacific Ocean, and does not apply in the Indian Ocean. Low‐level wind in boreal summer is primarily westerly in the TISO region and both MJO and MISO prefer warm SST. In another study, Kanemaru and Masunaga (2014) used various satellite data products by applying composite analysis with TISO phases to show that the basic westerly background surface wind is important for the warm SST anomaly ahead of TISO convection. Adames et al. (2016) found that the amplitude of MJO‐related convection, column‐integrated moisture, and low‐level zonal wind are stronger in the summer hemisphere. Hazra and Krishnamurthy (2017) compared MJO and MISO composites based on three‐dimensional diabatic heating and found that MJO and MISO are driven by the same mechanisms. They hypothesized that the difference in meridional propagation of TISO is due to the meridional shift in differential heating between two seasons. It seems that the interhemispheric annual cycle migration of the maximum of mean background SST, moisture, and low‐level zonal wind are important in the seasonality of TISO. It is convenient to assume that SST is the main driver for the seasonality of TISO. But the relative roles of SST, low‐level moisture, and surface wind have not been well explored. It is not clear how and to what extent the seasonal variations in TISO and its mean background state are related.

Although both components of TISO (MJO and MISO) share a common spectral peak, MJO and MISO events largely occur in different seasons, at different latitudes, and are governed by different mechanisms. MJO and MISO also have a different phase speeds and directions of propagation. Most of the commonly used diagnostics to understand TISO consider dimensional reduction by seasonal and spatial averages or decomposition into basis functions that maximize the variance or other characteristics (e.g., empirical orthogonal functions). This approach has the disadvantage that some information regarding direction of propagation, location, and phase speed may be lost. As an alternative, we examine TISO by tracking each event and compositing events on the basis of direction of propagation. A similar approach was adopted by Wang and Rui (1990)b; hereafter WR90) and Jones, Waliser, et al. (2004). In their study WR90 tracked the temporal evolution of pentad mean Outgoing Longwave Radiation (OLR) anomaly from 1975–1985 (excluding 1978) from the National Oceanic and Atmospheric Administration (NOAA), with objective criteria, e.g., a life span of at least four pentads, width exceeding 30° in longitude over the lifetime of an event, and a central minimum OLR less than −15 W/m^2^ over the lifetime of an event and less than −25 W/m^2^ during the strongest stage of evolution. WR90 identified 122 events, classified into three categories: (a) eastward moving from Africa to the mid‐Pacific Ocean, (b) initially eastward along the equator then turning north‐east or south‐east, (c) a split center with the main anomaly moving eastward along the equator and simultaneously a secondary center moving northward over the Indian or western Pacific Ocean. Independent northward propagation (northward only, not eastward) occurs in boreal summer during May–October. WR90 composited a synoptic climatology of TISO (which they referred to as tropical intraseasonal convective anomalies) and suggested that the dynamic effect of the equator and the thermal effect of the underlying warm water is responsible for TISO in deep tropics. The annual march of the thermal equator and its interaction with the mean monsoonal circulations are the main contributing factors to the meridional propagation of TISO events. However, because of the limited number of years (1975–1985) analyzed, WR90 were not able to draw any statistical inference from their analysis. Jones, Waliser, et al. (2004); hereafter J04) updated the climatology of eastward moving TISO events by using 24 years of OLR data with a more sophisticated tracking algorithm. They objectively divided TISO events into three categories according to their propagation characteristics: (a) originating in the Indian Ocean and dying in in the Indian Ocean after propagating eastward; (b) MJO events; and (c) summer ISO events. Based on their analysis of the spatial and temporal characteristics of TISO events with the longer time series of data, J04 concluded that MJO events have a longer life span, greater intensity, and larger variability than ISO events. The algorithm developed by J04 uses spatial correlation to connect identified OLR contiguous regions. When MJO crosses the Maritime Continent, over the land, MJO weakens and shrink in size. TISO‐related convective activity moves erratically from one frame to another. Therefore, it is possible that spatial correlation between two continuous OLR contiguous regions become lower than threshold and event can be end prematurely.

Zhang and Ling (2017) used positive precipitation anomalies as a variable in tracking eastward propagating TISO (MJO) event along the equator. MJO events in this method are identified on the basis of best known characteristics of MJO, such as propagation and time scale. Though this required average of filtered precipitation anomalies over the latitudinal belt (15°S–15°N) but provide some useful properties of an identified MJO events such as starting and ending longitudes, propagation range, strength in terms of precipitation, and life span, which are not available from MJO indices based on empirical orthogonal functions. Simultaneous tracking of eastward and northward propagating TISO is not possible from the methods that include any regional averaging of TISO signal. Kerns and Chen (2016) developed a large‐scale precipitation tracking method to track precipitation and convection related to eastward propagating TISO (MJO) in two‐dimensional space and time. Large‐scale precipitation tracking method uses spatially smoothed 3‐day accumulated total precipitation to identify and track large‐scale precipitation feature related to MJO. Using 17 years of Tropical Rainfall Measurement data large‐scale precipitation method, it identified 42 eastward propagating MJO events and noticed 75% MJO events that crosses Maritime Continent had significant MJO signature in Real Time Multivariate MJO (RMM) while only 33% non‐Maritime Continent crossing event occurred in RMM MJO signal. A discrepancy between two tracking methods is due to a limitation of RMM and other EOF‐based methods that is heavily weighted toward upper 200 hPa zonal wind signal (Straub, 2013).

The objective of this study is to understand the relative roles of SST, moisture, and atmospheric circulation variability in preferred geographical regions with respect to the propagation characteristics of TISO. To avoid any presumption about defining seasons and to understand the relationship between TISO and the continuously moving mean background state, it requires a tracking method that can simultaneously track eastward propagating TISO and northward propagating TISO in Indian Ocean and west Pacific Ocean into two‐dimensional space and time. We introduce a new feature tracking method to identify and track tropical intraseasonal convective anomalies. Tracks of objectively identified TISO will be used for this analysis. This algorithm is similar to the tracking method of J04 in terms of feature identification, but it is less restrictive in its classification of TISO events. This method has been applied to data with higher temporal resolution, and it provides a more accurate way of objectively identifying propagating, coherent features. It also provides additional characteristics of propagating convective features such as size, intensity, geographical location of propagation, phase speed, life span, regions of initiation and dissipation, and their seasonal and interannual variability. This paper will be organized as follows: Data set and procedure of algorithm development will be described in section 2. Characteristics of eastward and northward propagating intraseasonal convective envelopes will be discussed in section 3. Relationship between TISO and mean background state will be discussed in section 4. Description of numerical experiments and results will be presented in section 5, and section 6 will provide discussion and conclusions. A companion paper to this study uses tracks from this algorithm to understand the dynamics of the TISO from a Lagrangian perspective, diagnostic of moist static energy budget of MJO provides a useful insight to TISO life cycle.

## 2 Data and Methods

### 2.1 Observational Data

To identify and track TISO events, 39 years (1979–2017) of OLR (https://www.esrl.noaa.gov/psd/data/gridded/data.interp_OLR.html) data (Liebmann & Smith, 1996) from NOAA (2.5° × 2.5° grid) are used, because anomalous OLR is considered a proxy for large‐scale tropical convective anomalies (Waliser et al., 1993; K. Lau & Chan, 1986). Daily OLR anomalies (OLRA) are obtained by removing the mean and three annual harmonics from the data at each grid‐point. The data are band‐pass filtered using a Lanczos filter in the 20–100 days' band to obtain intraseasonal anomalies. Lanczos filter (Lanczos, 1956) uses sigma factor and more effectively reduces amplitude of Gibbs oscillation; therefore, it effectively isolate the intraseasonal time scale variability as compared to running mean, Fast Fourier transform, and Gaussian filters. Finally, the filtered OLRA are spatially smoothed using a 9‐point weighted average. For the life cycle analysis of each kind of TISO, variables including wind and specific humidity are obtained from the ERA‐Interim reanalysis (Dee et al., 2011). For SST, the OISST v2 weekly data (Reynolds et al., 2007) between 35°S and 35°N are interpolated to daily values.

### 2.2 Methodology to Identify and Track TISO

#### 2.2.1 Tracking Algorithm

A new algorithm based upon the Multiple Object Tracking (MOT) method was developed to identify, track, and classify TISO events based on their direction of propagation. MOT is widely used in computer vision for locating multiple objects, maintaining their identities, and getting their trajectories from a given input video. MOT is used to track pedestrians in streets, vehicles, players on the court, and other moving objects (e.g., Granstrom et al., 2016). If each daily map of filtered OLR anomalies is considered as analogous to an image from a static camera, then a sequence of images from daily filtered OLR anomalies can be considered as analogous to video of convective anomalies over the tropics. Therefore, tracking of mesoscale convective anomalies is possible with the MOT method; however, there are a few challenges. Because convective cloud systems (CCSs) can change their size and shape continuously from day to day (frame to frame), it can be hard to preserve their identities, but it is possible to track their centroid position. CCSs can originate or die in each frame; therefore, the number of CCSs does not remain the same during tracking. A systematic framework using a motion‐based MOT algorithm (Li et al., 2010) has been developed to identify, track, and classify TISO events. We have used size and intensity threshold criteria on the basis of well‐observed characteristics of TISO, which is similar to WR90, J04, and Kerns and Chen (2016). The OLRA data from 35°N–35°S are used to track every individual TISO event. OLR minima below −15 W/m^2^ are considered to serve as a proxy of large (~10^5^ km^2^) CCSs. The tracking algorithm consists of identifying, tracking, and classifying CCSs.

#### 2.2.2 Identification and Analysis of CCSs

**Figure 1:**
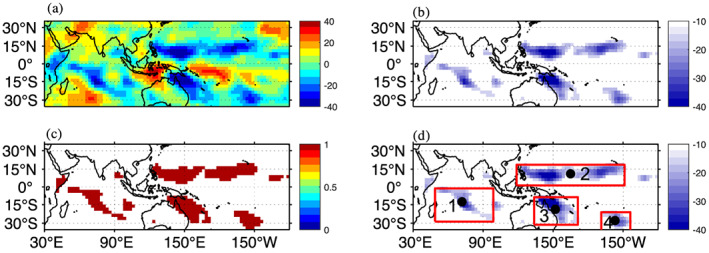
(a) 20–100 days band‐pass filtered and smoothed OLRA for the 12th of April 1979, (b) OLR anomaly same as (a) but masked for values less than −15 W/m^2^. (c) OLR anomaly converted into binary image values less than −15 W/m^2^ are set equal to 1 and other pixel are set is 0, (d) identified and labeled cloud system after image analysis.

Identification of CCSs requires localizing them into a single image. Daily OLR maps can be considered a series of global images from a stationary camera. To localize CCSs at the intraseasonal time scale from the intraseasonally filtered and spatially smoothed OLRA, we used the same criteria as in the manual method. For example, Figure 1a shows the filtered and smoothed OLRA for the 12th of April 1979. By masking out any filtered OLRA greater than −15 W/m^2^ per the magnitude threshold criteria of WR90, all CCSs around the globe between 35°S and 35°N become apparent. The filtered OLRA maps are next converted into binary images to conduct blob analysis (Figure 1c). While the CCSs are visually apparent in Figure 1b, additional quantitative analysis of spatially coherent objects blob analysis is needed. Any group of connected pixels is referred to as a blob. Blob analysis comprises blob extraction and classification. Blobs are extracted by using 8‐connectivity. After blob extraction, each blob is analyzed to retrieve information about blob size (number of pixels), equivalent diameter, mean intensity (magnitude of negative OLR anomaly), centroid (mass weighted), bounding box, orientation, major and minor axis lengths, and frame number. These data are stored for each frame.

The quantitative characteristics of each blob can be used to decide whether it represents a system of convective clouds. Here we denote a blob as a CCS if it has size greater than 15 grid‐points (10^5^ km^2^). Figure 1d shows all selected CCSs on the 12th of April 1979 enclosed in their bounding boxes and numbered. Some small CCSs can be neglected by using the size threshold criteria. Tracks are initialized for each labeled CCS and information regarding each convective system is saved in the corresponding track. OLR anomalies corresponding each CCS are projected over analyzed image.

#### 2.2.3 Tracking of CCSs

Tracking localized CCSs requires maintaining their identities from frame to frame. Tracking a moving object requires a motion model so that its position in future frames can be predicted to reduce the search space and time. We have used a Kalman filter approach with a constant acceleration motion model, and we assume that convective anomalies move smoothly from one frame to the next. The Kalman filter is an estimator that is used to predict and correct the state in various linear processes (Kalman, 1960). The tracking algorithm using a Kalman filter can be divided into three parts:

**Motion model**: A CCS can be represented by its centroid, because the size of the CCS changes from frame to frame. For any given frame, a state vector can be defined for the position and velocity the centroid of a CCS as follows:

(1) $$X_{k}={\left[x_{k}\;y_{k}\;u_{k}\;v_{k}\right]}^{T}$$

Here x_k_ and y_k_ are position coordinate, u_k_ and v_k_ are velocity components in longitudinal and latitudinal directions, respectively. For all the tracks, at initial time step velocity is initialized as zero. The position of each CCS in frame k + 1 can be predicted from frame k as follows:

(2) $$X_{p\left(k+1\right)}=AX_{k}+w_{k}$$

Here X_p (k+1)_ is a predicted state in frame k+1 and w_k_ is process noise with Gaussian distribution as follows:

(3) $$P\left(w\right)\sim N\left(0,Q\right)$$

A represents the state transition matrix and for a given state vector it will be

(4) $$A=\left[\begin{array}{cccc} 1 & 0 & \mathrm{dt} & 0\\ 0 & 1 & 0 & \mathrm{dt}\\ 0 & 0 & 1 & 0\\ 0 & 0 & 0 & 1 \end{array}\right]$$

The time and sampling intervals for convective anomalies are both 1 day, so dt = 1. The variance of the predicted state is given as

(5) $$P_{k+1}=AP_{k}A^{T}+Q\left(w\right)$$

For every frame, the position of each CCS is measure by its centroid position; therefore, the measurement vector can be written as

(6) $$Z_{k}={\left[\begin{array}{cc} x_{k} & y_{k} \end{array}\right]}^{T}$$

Then the measurement equation for frame k+1 will be

(7) $$Z_{k+1}=HX_{k}+s_{k}$$

Here Z_k+1_ is the measured position in frame k+1, s_k_ is measurement noise with a Gaussian distribution as follows:

(8) $$P\left(v\right)\sim N\left(0,Q\right)$$

H represents the state measurement matrix and for a given state vector it will be

(9) $$H=\left[\begin{array}{cccc} 1 & 0 & 0 & 0\\ 0 & 1 & 0 & 0 \end{array}\right]$$

#### 2.2.4 Connecting CCS

Before we update the predicted position of the centroid of a given CCS using a linear combination of the predicted and measured states in frame k+1, it is necessary to establish a connection between the predicted and measured CCS. Because CCS shape changes continuously, it is hard to maintain their identities with shape and size. Therefore, to connect CCS from frame to frame, we use two parameters: (a) the position (coordinates) of the centroid and (b) a critical search radius (D_c_). The critical search radius forms an imaginary search circle around the predicted CCS centroid. If a measured CCS is found in this circle, it can be connected to predicted CCS. If a CCS is not found in this search space, the predicted CCS is declared dead and the corresponding track is closed. D_c_ can be defined based on our prior knowledge about the speed of the moving object and the spatiotemporal resolution of data. Because the propagation speed of TISO is ~1–5 m/s (Sikka & Gadgil, 1980; Zhang, 2005), D_c_ is chosen as 6 grid points to capture TISO moving with speeds from 1–10 m/s.

First, we have calculated distance of each measured CCS from a predicted CCS in frame k+1. Let we have n predicted CCS from frame k and m measured CCS in frame k+1. Each predicted CCS is labeled with a number from 1 to n and each measured CCS is labeled with number from 1 to m. The distance of each predicted CCS from each measured CCS is given as

(10) $$D_{\mathrm{ij}}=\sqrt{{\left(x_{p\left(k+1\right)}^{i}-x_{k+1}^{j}\right)}^{2}+{\left(y_{p\left(k+1\right)}^{i}-y_{k+1}^{j}\right)}^{2}}$$

Here D_ij_ is a matrix of size m × n. Each row of matrix D_ij_ represents the predicted CCS and each column represents the measured CCS. Element d_ij_ represents the distance between the *i*th predicted and *j*th measured CCS. Row minima of D_ij_can be written as

(11) $$u_{j}=\min_{j\in j}D_{\mathrm{ij}}\;i=1\ldots \;n$$

The index of a row minimum of D_ij_ and the minimum distance (u_j_) between predicted CCS and measured CCSs is used to connect predicted and measured CCS as follows. For given *i*th predicted CCS

(12) =uj≤Dc,Xpi∈Zjuj>Dc,Xpiis dead

If any measured CCS is not assigned to any predicted CCS, then that CCS is considered as new CCS. A new track for unassigned CCS is initialized, this criterion will hold for all new CCSs at each time step.

#### 2.2.5 Model Updating

After the process of CCS association, the state of the associated CCSs is updated with a linear combination of predicted and associated (measured) CCS in the update step of the Kalman filter as follows:

(13) $$X_{k+1}=X_{p\left(k+1\right)}+K_{k+1}\left(Z_{k+1}-HX_{k+1}\right),$$

(14) $$K_{k+1}=P_{k}H^{T}{\left({\mathrm{HP}}_{k}H^{T}+R\right)}^{-1},$$

(15) $$P_{k+1}=\left(1-K_{k+1}H\right)P_{k}$$

The updated centroid positions along with other information from image analysis are saved in the corresponding track for frame k + 1. New tracks with new label numbers are initialized for each new CCS from frame k+1. The labels corresponding to dead tracks are removed and tracks closed. The same recursive process is repeated for each successive frame. Algorithm keeps track of all the CCS that has area greater than 10^5^ km^2^, if one track splits into two tracks at a given frame then algorithm considers the nearest CCS as older track and a new track is initiated for splitted CCS. Two splitted tracks that belong to the same track can be easily identified during post processing of identified tracks, as track corresponding to each CCS is initiated and saved. Merging of the tracks can happen during tracking, two tracks are considered as merged when two projected CCS from frame k are associated to the same observed CCS in k + 1, algorithm considers it case of merging, algorithm gives preference to a track that originated earlier and the CCS that initiated later is declared dead and corresponding tracks is closed. In the following, the method described above is referred to as the MOT algorithm. MOT algorithm is applied over the region 0° to 240°E and 35°S to 35°N over the period of 1979–2017. The tracks originated between 0° to 160°E and 20°S to 20°N and life span greater than 15 days are retained for further classification.

### 2.3 Classification

The identified tracks are classified based on their direction of propagation. The direction of propagation is obtained by considering the angle with the horizontal (west to east) axis made by the vector passing from the starting to the ending point of a track. Tracks are first classified into eight ordinals based on the direction of propagation as follows: eastward (337.5°–22.5°), northeastward (22.5°–67.5°), northward (67.5°–112.5°), northwestward (112.5°–157.5°), westward (157.5°–202.5°), southwestward (202.5°–247.5°), southward (247.5°–292.5°), and southeastward (292.5°–337.5°) In order to track the two dominate modes of TISO (MJO and MISO), eastward and northward propagating TISO events are classified separately. An event is classified as eastward moving if

- the track angle lies in the eastward ordinal;
- or lies in the southeastward ordinal and track has life span at least 15 days in deep tropics (15°S–15°N);
- or lies in northeastward ordinal and tracks has life span at least 15 days in deep tropics and track dose not crosses 15°N, if dies before 110°E; and
- CCS moves at least 30°E after initiation.

An event is classified as northward moving if

- the track angle lies in the northward ordinal;
- or track angle lies in northeastward ordinal, dissipated before 110°E and crosses 15°N;
- or track angle lies in northwestward ordinal and crosses 15°N; and
- CCS moves at least 15°N after initiation.

### 2.4 Track Association

**Figure 2:**
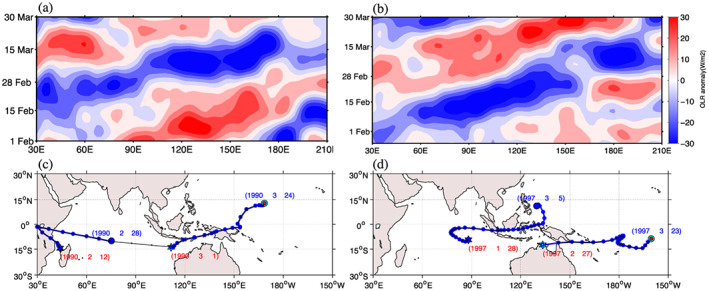
(a) Hovmöller diagram of 20–100 days band‐pass filtered and smoothed OLRA for the 1st of February to the 30th of March 1990, (b) Hovmöller diagram of 20–100 days band‐pass filtered and smoothed OLRA for the 25th of January to the 30th of March 1997, (c) tracks of two eastward propagating events identified by algorithm (blue), connection of these two tracks (black), (d) tracks of two eastward propagating events identified by algorithm (blue), connection of these two tracks (black).

For some events, after initiation in Indian Ocean, an eastward propagating TISO event may reach the Maritime Continent and weaken to the extent that the algorithm declares it dead. A similar behavior was noted by Kerns and Chen (2016): while the evolution of a given, large‐scale feature seems continuous, the centroid can move erratically from day to day. To make a connection between tracks that belong to same TISO event, the algorithm was applied to each identified track for 30 days before and after. The algorithm checks whether any short track of at least 10 days lies within 30° radius and start or end within 10 days from the starting or endpoint of the given track, and, if so, the two identified tracks are connected at the day of minimum distance and this connected track replaces the original track. Figure 2a is Hovmöller diagram of 20–100 days band‐pass filtered OLRA for a particular period in early 1990. The figure shows an eastward propagating TISO event that was initiated at 30°E on the 15th of February and propagated to the west Pacific Ocean, ending on the 20th of March. The corresponding tracks detected by the MOT algorithm are shown in Figure 2c: the MOT algorithm correctly detected the initiation of the event, but it lost that track around 80°E and identified another track that was initiated at 115°E, despite the fact that it was a single event (Figure 2a). A similar example is shown in Figure 2b in early 1997, with an eastward propagating TISO event that was initiated in the Indian ocean around 60°E in late January and died after crossing the date line in late March. The Hovmöller diagram (Figure 2b) suggests that the event crossed the Maritime Continent without any loss of intensity, while, in fact, the CCS split into two centers over the Maritime Continent and the MOT algorithm started tracking them separately (Figure 2d). The second pass of the MOT algorithm identified the separate tracks in both cases as belonging to the same TISO events and joined them as a single track. After the track association step, eastward moving tracks that cover more than 50° longitude and live for more than 20 days after initiation are considered eastward propagating TISO events.

## 3 Results

### 3.1 Average Characteristics of TISO

The MOT algorithm described in section 2 was applied to the same data but for the period 1979–2017 between 35°S–35°N and 0–240°E. All tracks identified by the MOT algorithm were classified according to their direction of propagation as discussed in section 2.3. Table 1 summarizes the results of this classification. As shown in Table 1, the MOT algorithm identified 90 eastward‐ and 102 northward‐moving TISO events, totals that are quite close to the number of manually identified TISO events in both classes. The average speed is calculated by calculating the distance between two successive centroid locations and averaging over the lifetime of each event. Because the centroid locations can change erratically from day to day, all tracks were smoothed over three successive days before calculating the phase speed. The average speed for eastward‐moving TISO events is 4.1 m/s, with an average duration of 44 days. Northward‐moving TISO events propagate at an average of 2.7 m/s and last an average of 22 days before dying in high latitudes. Previous studies have found that TISO events propagate northward at 1° day^−1^ (about 1.3 m/s) during boreal summer (W. K.‐M. Lau & Waliser, 2012; Sikka & Gadgil, 1980), while this analysis shows northward propagation speed 2.7 m/s using the algorithm. The phase speed shown here is different from previous studies, because conventionally the eastward (northward) propagation speed is calculated from the slope of the time‐longitude (time‐latitude) diagram of the propagating signal, which is basically a longitudinal (latitudinal) displacement rate. In contrast, the MOT algorithm tracks the movement of propagating CCS, which sometimes includes both eastward and northward propagation. Which is a total speed of the event and can be broken down eastward and northward (longitudinal and latitudinal) displacement component. In MOT algorithm, latitudinal displacement rate is found equivalent to 1.3 m/s, and a longitudinal displacement rate equivalent to 3.8 m/s, which agrees with previous studies. Both the speed and average life span of events detected by the MOT algorithm are in good agreement with the manual analysis and results reported in previous studies (WR90; J04). J04 found 110 events in 24 years (approximately five events per year) using an objective method, which is quite comparable to the 192 events in 39 years of data (approximately five events per year) found using the MOT algorithm described herein. WR90 reported finding 122 events in 10 years of data (∼12 events per year), which is much higher than the detection rates of J04 and the present study. Large‐scale precipitation tracking method developed by Kerns and Chen (2016) found 42 eastward propagating cases in 18 years of data (2.5 events per year), which is similar to that found using the MOT algorithm, taking into account the fact that Kerns and Chen (2016) applied tracking only for October–March every year.

**Table 1:** Average Characteristics of Tropical Intraseasonal Oscillations

Sr. No	Class	Events	Ave. speed (m/s)	Ave. life span (days)
1	Eastward	90	4.1	44
2	Northward	102	2.7	22

### 3.2 Verification of TISO Events

**Figure 3:**
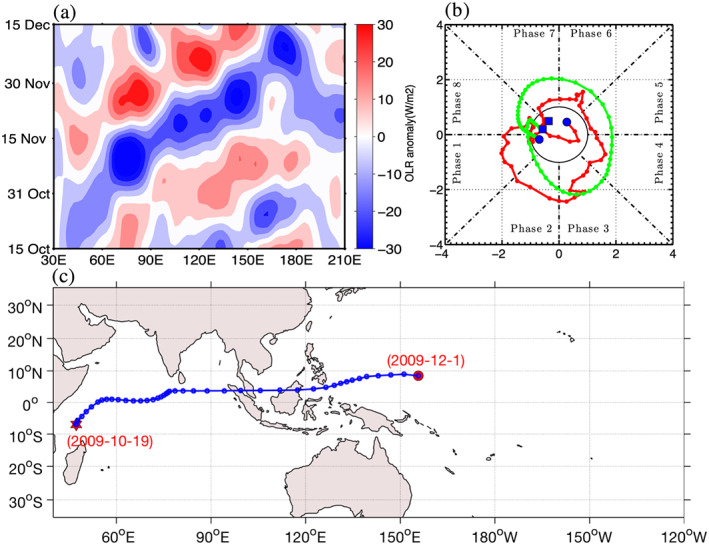
(a) Time longitude diagram of 20–100 days' band‐pass filtered OLRA averaged between 15°S–15°N the 15th of October 2009 and the 15th of December 2009. (b) Phase diagram of RMM (red) and ROMI (green) for similar period, blue filed square represents starting date and blue filled circle represent last date. (c) Eastward propagating TISO event identified by MOT algorithm, initiation (red star), dissipation (red circle).

TISO events identified by the MOT algorithm are evaluated with well‐observed MJO activity during the Year of Tropical Convection (Moncrieff et al., 2012) and Dynamics of Madden Julian Oscillation (DYNAMO; Yoneyama et al., 2013). A well‐defined MJO event occurred during the Year of Tropical Convection period in late October–November 2009. The algorithm was applied 5 days beyond the lifespan of each well‐documented TISO event. Figure 3a shows 20–100 days filtered OLRA averaged between 15°S and 15°N corresponding to an eastward propagating TISO event that occurred between the 15th of October 2009 and the 15th of December 2009. In that event, the eastward propagation was initiated on the 25th of October at 45°E, and dissipated after crossing 150°E. Westward propagating OLRA is also evident in the Hovmöller diagram. Figure 3b shows the phase space diagram. The RMM (Wheeler & Hendon, 2004) index and the Real time OLR‐based MJO index (ROMI; Kikuchi et al., 2012) for the same period as in Figure 3a. Both RMM and ROMI indicate initiation of an MJO event around the 25th of October, although RMM leads ROMI by a couple of days, and both indices capture the propagation of this event into the western Pacific Ocean. The TISO event identified by the MOT algorithm is shown in Figure 3c, agreeing quite closely with the initiation dissipation longitudes and dates from the indices that are used in real time MJO monitoring. The MOT algorithm can provide some other information about the size and intensity of OLRA on each day (not shown), characteristics that are not obtained with conventional MJO monitoring methods.

**Figure 4:**
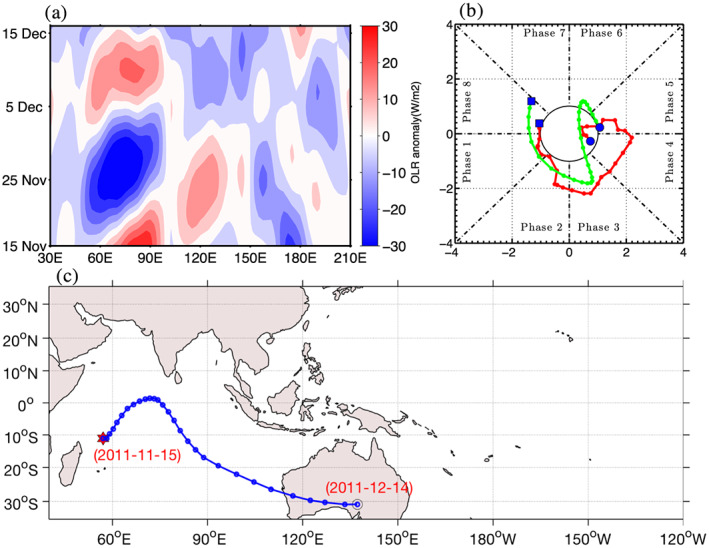
(a) Time longitude diagram of 20‐100 days' band‐pass filtered OLRA averaged between 15°S–15°N for the 15th of November 2012 and the 15th of December 2012. (b) Phase diagram of RMM (red) and ROMI (green) for similar period, blue filled square represents starting date and blue filled circles represent last date. (c) Eastward propagating TISO event identified by MOT algorithm, initiation (red star), dissipation (red circle).

Figure 4 shows an eastward propagating TISO event identified between the 15th of November 2011 and the 15th of December 2011, an event documented as MJO‐2 in the DYNAMO experiment. The Hovmöller diagram shows that the event started at 60°E on the 18th of November, and the MOT algorithm (Figure 4c) captures both the initiation date and longitude. Both RMM and ROMI indices show initiation in phase 1 (Indian Ocean), with initiation in RMM leading ROMI by a few days. The phase lag between these two indices was also noticed in Kiladis et al. (2014) during the September–November season. While moving eastward, the OLRA grows in intensify until it reaches at 90°E (Figure 4a), after which the event decays in size and intensity, turning south‐westward and decaying over Australia. Kerns and Chen (2016) showed that MJO‐2 crosses the Maritime Continent and decays to its northeast. Difference between two tracking method is because Kerns and Chen (2016) used a method that tracks total precipitation, which include variability of all time scale, while MOT method track convective envelope of 20–100 days band‐pass filtered OLRA. Gottschalck et al. (2013) noticed that in its later stage MJO‐2 become convectively coupled Kelvin wave, which may explain the discrepancy between the 3 days accumulated precipitation centroid. Amplitude of ROMI is rapidly decayed in phase 3 of this event, while the RMM maintains its high amplitude until phase 5, which is due to large loading of circulation component on RMM (Kiladis et al., 2014).

**Figure 5:**
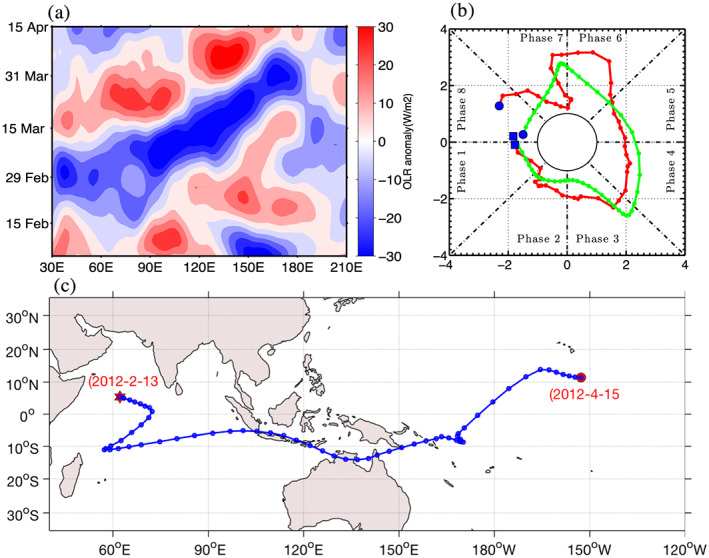
(a) Time longitude diagram of 20–100 days' band‐pass filtered OLRA averaged between 15°S–15°N for the 10th of February 2012 and the 15th of April 2012. (b) Phase diagram of RMM (red) and ROMI (green) for similar period, blue filed square represents starting date and blue filled circle represent last date. (c) Eastward propagating TISO event identified by MOT algorithm, initiation (red star), dissipation (red circle).

Figure 5 shows an eastward propagating TISO event identified between early February and early April 2012 during the DYNAMO period. This event is documented as MJO‐4 in Yoneyama et al. (2013). The event was initiated on the 18th of February at 60°E, initially propagated westward while increasing in size, then propagated eastward for the rest of its life span. This event was so strong that it crossed the Maritime Continent without any intensity loss, eventually dying at 180°W in early April, as shown in Figures 5a and 5b. The RMM and ROMI both maintained high amplitude (≥1) during the entire life span of MJO‐4, from phase 1 to phase 8. It is interesting to note that for stronger TISO events, both RMM (circulation and OLR based) and ROMI (only OLR based) indices agreed in terms of event initiation, dissipation, and life span. The MOT algorithm successfully detected and tracked MJO‐4 in agreement with RMM and ROMI.

**Figure 6:**
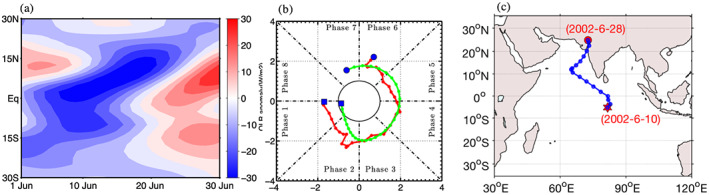
(a) Time latitude diagram of 20–100 days' band‐pass filtered OLRA averaged between 60–100°E for the 10th of June 2002 and the 30th of June 2002. (b) Phase diagram of RMM (red) and ROMI (green) for similar period, blue filed square represents starting date and blue filled circle represent last date. (c) Eastward propagating TISO event identified by MOT algorithm, initiation (red star), dissipation (red circle).

Figure 6a showing a northward propagating track of a TISO event identified between the 10th of June 2002 and the 30th of June 2002. This TISO event was initiated in the southern hemisphere around 15°S and propagated toward the Arabian Sea and dissipated in northwestern India in late June after crossing 20°N. The time‐latitude diagram of OLRA also shows that a slow northward propagating event initiated near 15°S and the event died in late June after crossing 15°N. The RMM and ROMI indices also agree with northward propagation over same period, both RMM and ROMI amplitude remain above one from phase 1 to phase 8. As we have seen the MOT algorithm successfully detected and tracked some very well observed TISO events.

The MOT algorithm closely agrees with ROMI in terms of initiation, dissipation, and life span of the event as compared to RMM, because both MJO identification methods use similarly processed satellite data, but the MOT algorithm additionally provides information about the spatiotemporal evolution in two‐dimensional space, which is not possible with the EOF‐based tracking methods. MOT tracking algorithm is less sensitive to the choice of subjective parameter during mature stage of propagation. While initiation, dissipation date, and location are sensitive to the choice of intensity threshold by 2–7 days and 10°–15°, respectively (Figure S1). MOT algorithm is less sensitive to the choice of search radius and object size threshold in terms of initiation, dissipation date, and location.

### 3.3 Spatial and Seasonal Characteristics of the TISO

**Figure 7:**
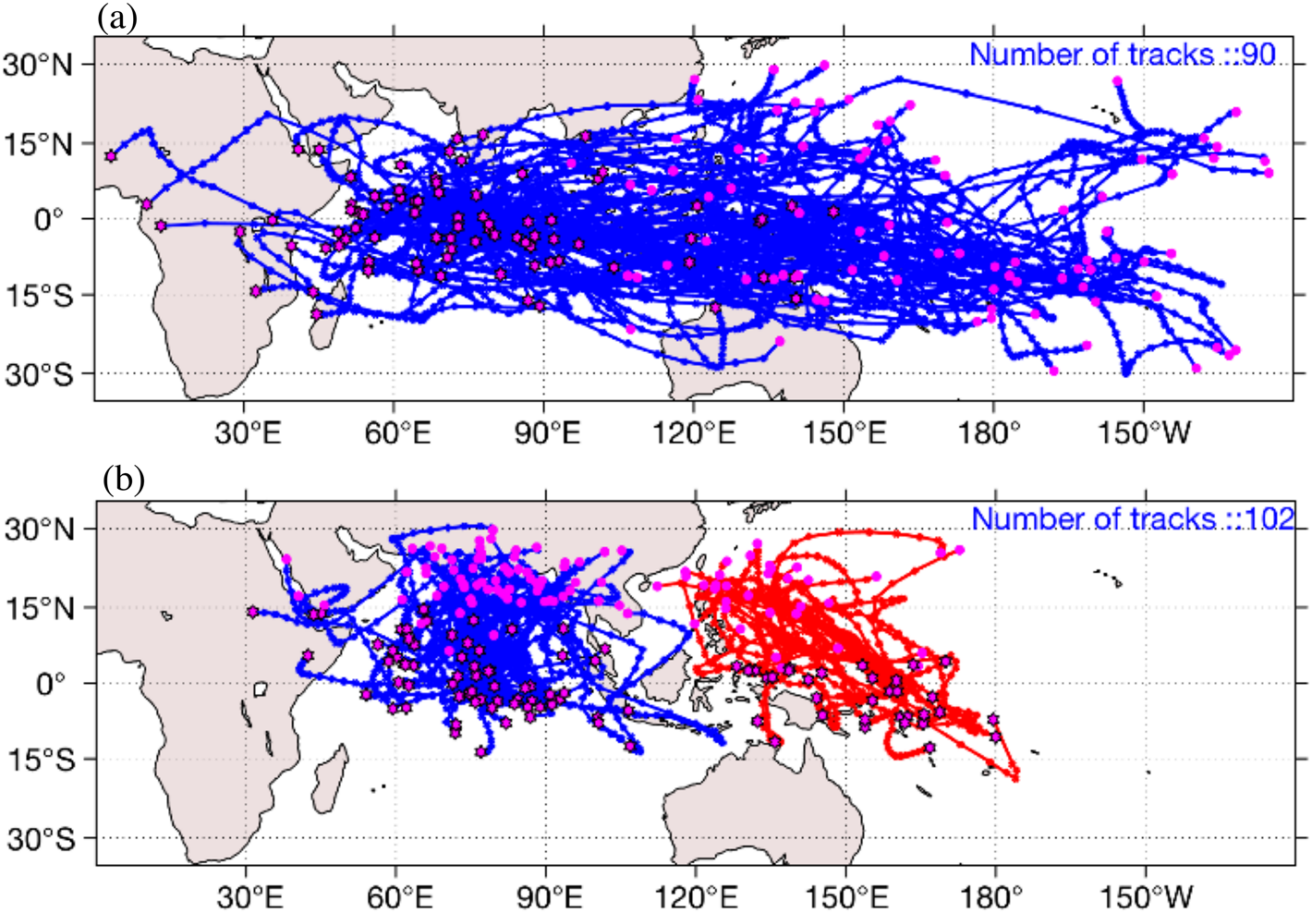
(a) Tracks of objectively identified (MOT algorithm) eastward propagating TISO events from the period of 1979–2017. (b) Tracks of objectively identified (MOT algorithm) northward propagating TISO events from the period of 1979–2017. Initiation locations are shown by magenta asterisk and dissipation location are represented by magenta circles.

The tracks of eastward and northward propagating TISO events from the MOT algorithm are shown in Figure 7. Most eastward‐moving tracks are found south of the equator between 5°N–15°S, beginning in the western to central Indian Ocean and propagating eastward or northward. After crossing the Maritime Continent, eastward‐moving TISO events turn southward over the South Pacific Convergence Zone. Northward propagation can be seen in both of the ocean basins. Two types of northward propagation happen in the Indian Ocean sector: moving northward immediately after initiation and (b) moving eastward at first, then turning northward. TISO events originating in the Pacific Ocean sector propagate directly northward only. After initiation, northward propagating TISO events die after crossing 20°N. The MOT algorithm , WR90 and J04 agree in terms of geographical locations of occurrence, initiation, and dissipation.

**Figure 8:**
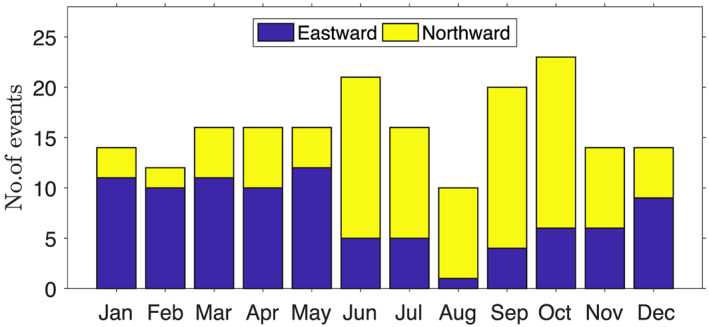
Seasonal frequency of eastward propagating and northward propagating TISO events.

Both eastward and northward propagating components of TISO exhibit a dependence on the phase of the annual cycle, a property referred to as seasonality. The seasonality of TISO events is measured as the fraction of TISO events that occur in each month of the year. The lifetime of a given TISO event may span multiple calendar months; therefore, the month corresponding to the midpoint date for each TISO event is considered the month of occurrence. The fractions of eastward and northward TISO occurrences for each month are shown in Figure 8. Eastward‐moving TISO events occur more often in boreal winter (DJFM:Dec.‐Jan‐Feb.‐Mar), but they can occur throughout the year (Figure 8a). Northward propagation is more common in boreal summer (MJJASO : May‐Jun‐Jul‐Aug‐Sep‐Oct), but sometimes occurs in November and December (Figure 8b). About 30% of TISO events are found outside of the conventional seasons of DJFM for eastward propagation and JJAS northward propagation. By using the conventional definition of seasons for MJO and MISO, a significant amount of TISO variability is unaccounted for. The results are also consistent with the previous studies of WR90 and J04, which also explored the similarities and differences between eastward and northward propagating TISO events. The MOT tracking method is appropriate to study the seasonality of TISO, because it can track TISO events without any assumption about season, which can leave some fraction of TISO‐related variance unaccounted for. Identifying TISO without the assumption of seasonality give us opportunity to understand the relationship between TISO and interhemispherically marching maxima in SST, moisture, and related circulation.

**Figure 9:**
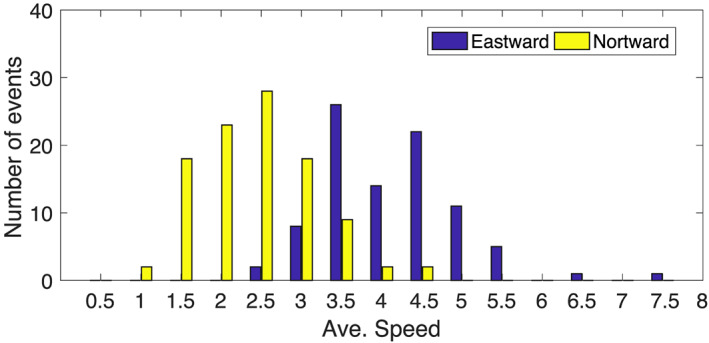
Average speed (m/s) of eastward propagating and northward propagating TISO events.

The average propagation speed of TISO events may vary from event to event and so, do their interactions with weather and climate around the globe. Recently, Yadav and Straus (2017) analyzed slow and fast propagating eastward propagating TISO events and their interaction with midlatitude weather. They found that midlatitude weather responds differently for slow and fast propagating MJO episodes. The histogram of eastward and northward propagating TISO events is shown in Figure 9. The average speed of eastward propagation ranges from 2.5 to 7.5 m/s, with most TISO events propagating 3.5–4.5 m/s and very few TISO events moving either very slowly (≤3 m/s) or very fast (≥5.5 m/s). The average speed of northward‐moving TISO events is 1.5–4 m/s. The MOT algorithm provides a systematic way to characterize TISO events based on slow and fast propagation.

**Figure 10:**
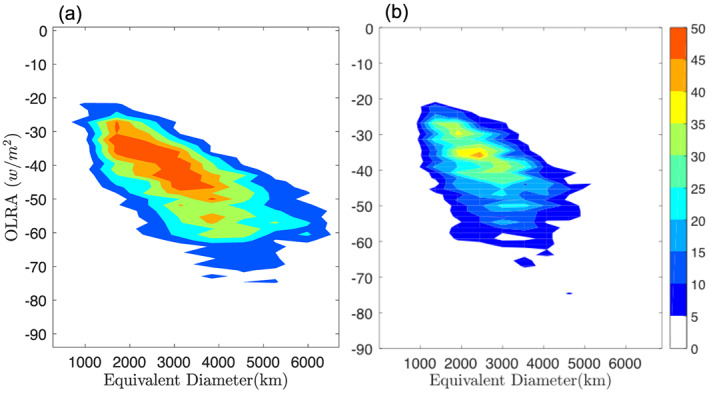
Two‐dimensional histogram of size and intensity (minimum OLR): (a) eastward propagating TISO events and (b) northward propagating TISO events. Color represents frequency of each histogram bin.

A two‐dimensional histogram of minimum OLR anomaly and equivalent diameter (defined as the diameter of circle that has equivalent area to the CCS, a parameter calculated during image analysis) of eastward events, northward events in the Indian Ocean, and northward events in the west Pacific Ocean are shown in Figure 10. The size of the CCS is directly proportional to the convective intensity (minimum OLRA). After initiation, as the CCS starts moving in an eastward or northward direction, it becomes bigger and more intense. The central minimum OLRA ranges from −20 to −70 W/m^2^ and the equivalent diameter varies from ∼1,000 to ∼5,000 km for eastward and northward propagating TISO events.

To analyze the statistics of the ensembles of identified tracks, we have used a spherical nonparametric kernel method (Hodges, 1996) to estimate track density. In this method, each data point (center of mass of each CCS) is assigned a probability density (kernel) function, and the kernel parameters that control the shape and amount of the smoothing are obtained by a data‐adaptive approach of cross validation. The track density is a function of the distribution of the data and the distribution of the estimation points. Track density is a unit‐less quantity and it is computed at each estimation point.

**Figure 11:**
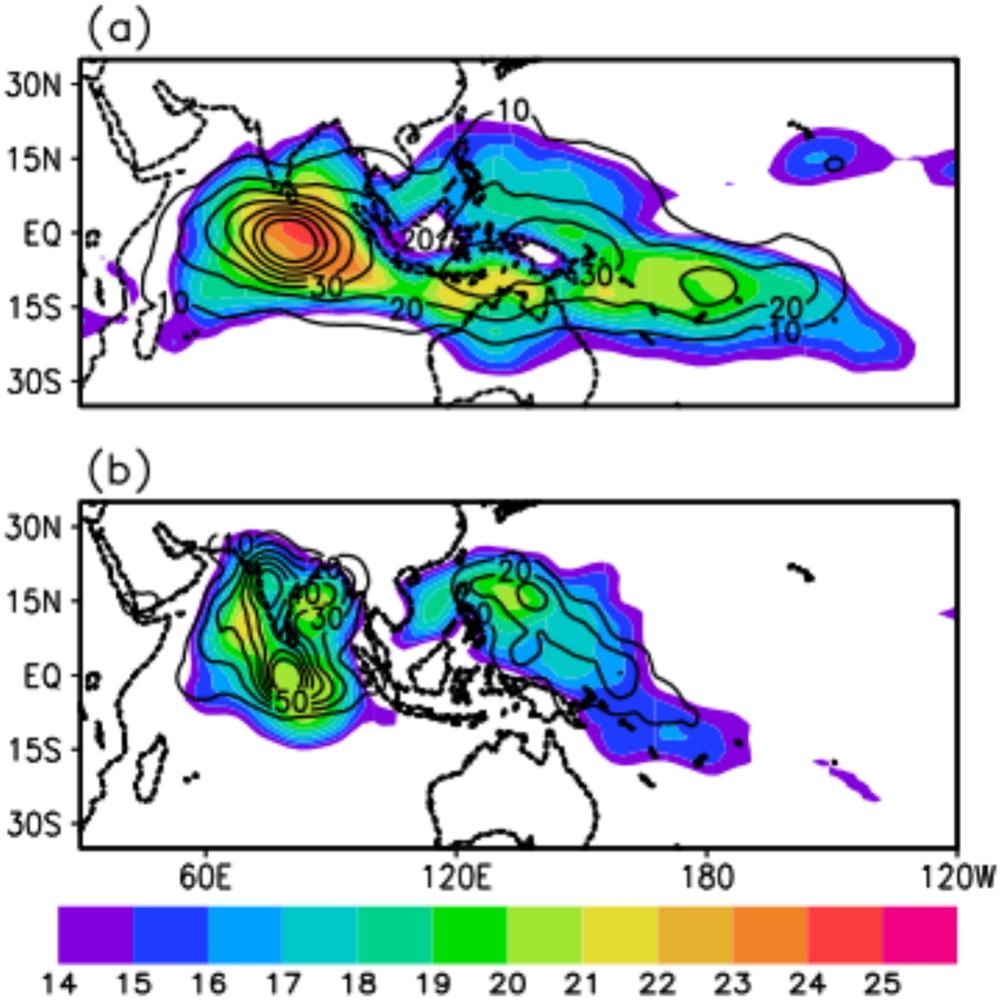
Standard deviation of 20–100 days' band‐pass filtered OLRA (shaded; w/m^2^) over the period of 1979–2017, track density (unitless), derived from the ensemble of tracks identified using MOT method (contours), (a) eastward propagating TISO events and (b) northward propagating TISO events.

Figure 11 shows the track density of the ensemble of tracks identified by using the MOT algorithm over the period 1979–2017. The centers of action of the intraseasonal variability, measured by the standard deviation of the intraseasonally filtered OLRA. We can see that the track density from the MOT algorithm (contours) and standard deviation of the intra seasonally filtered OLRA (shaded) are colocated, both track density and intraseasonal variability have maxima over the central Indian Ocean and west Pacific Ocean for the eastward propagating TISO events. The maxima of northward propagating TISO variability can be seen to align well with the maximum of track density of northward propagating TISO events. The intraseasonal variability of eastward and northward propagating TISO events is very well represented by track density. Therefore, track density, in addition to individual track statistics, can be used as a metric to construct composites to examine the mechanisms of the seasonal variability of TISO events.

## 4 Relationship Between TISO and Mean Background State

**Figure 12:**
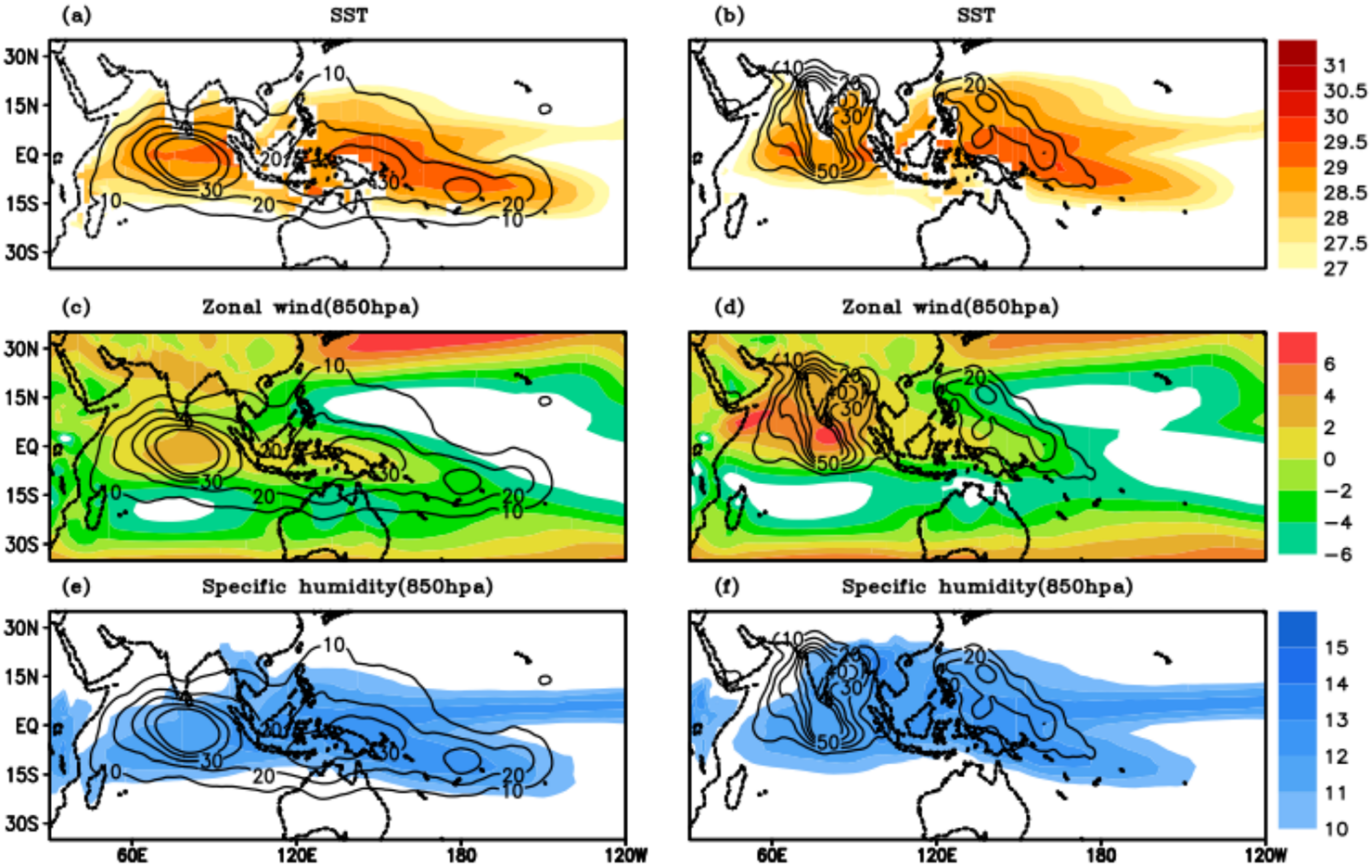
Composite of mean background state (shaded) (a) sea surface temperature, (c) zonal wind (850 hPa), and (e) specific humidity (850 hPa) with eastward moving TISO events tracks density (contours) and (b) sea surface temperature, (d) zonal wind, and (f) specific humidity with northward moving TISO events and tracks density (contours).

Figure 12 shows the composite of eastward‐ and northward‐moving tracks with SST, zonal wind at 850 hPa (U), and specific humidity at 850 hPa (q). All eastward‐moving tracks occur inside the 28 °C contour (over water warmer than 28 °C) and eastward propagation largely occurs in boreal winter, when the warmest SST is found in the Southern Hemisphere (Figure 12a). In boreal summer, when the warmest SST migrates to the Northern Hemisphere, TISO events are more likely to be northward‐propagating in both the Indian and western Pacific Ocean basins. Figures 12c and 12d show that both eastward‐ and northward‐propagating TISO events are more likely to occur in the region of westerly mean background wind. Similarly, q exceeds 10 g/kg in the area where TISO events are active in both hemispheres. The importance of SST and U was recognized in previous studies, e.g., Zhang and Dong (2004) examined the relative role of SST and the large‐scale circulation in the seasonality of MJO, while we can see that SST and U are important for both eastward and northward propagating TISO cases. Zhang and Dong (2004) also noticed that the seasonality in MJO is most pronounced in the western Pacific Ocean, less apparent in the eastern Pacific Ocean, and does not apply in the Indian Ocean. Composite analysis of objectively identified TISO events similarly shows that there is no seasonality in terms of SST migration in the Indian Ocean, but the region of westerly U migrates from the equator to 25°N. The simultaneous existence of warm SST and strong zonal wind north of the equator may be crucial for selective northward propagation in this region. It is interesting to note that in Figures 12b and 12d, while the SST in the western Pacific is sufficiently warm, the relatively weak U makes northward propagation less frequent as compared to its Indian ocean counterpart. The geographical location of TISO occurrences is coupled with SST, moisture, and lower tropospheric circulation. The seasonal migration of mean background state is a potential source of the seasonal characteristics of TISO. From the composites in Figure 12, we have hypothesized that the geographical location of TISO occurrences is coupled with SST, moisture, and lower tropospheric circulation. The seasonal migration of the mean background state is a potential source of the seasonal variation of TISO.

## 5 Numerical Experiments: Sensitivity of the TISO to Mean Background State of SST and Low‐Level Circulation

It is impossible to separate the effect of circulation and SST on TISO in nature but we can design numerical experiments to separate the effects of annual cycle mean SST from zonal wind to understand their relative importance for TISO dynamics. To test the above‐mentioned hypothesis, we have performed two sets of experiments in which SST and solar insolation is decoupled in order to separate mean SST and low‐level circulation. SP‐CAM4 coupled to model SpCCSM4 simulated monthly climatological SST is used for current study. Simulations will be carried out on the finite volume grid at 1.9° × 2.5° resolution. Each 2‐D cloud‐resolving model is embedded in the east‐west direction at 4‐km horizontal resolution with 30 vertical levels. The time step for the cloud model is 20s. The cloud‐resolving model runs continuously and shares information with the GCM (host model) at the host models time step. The subseasonal variability of tropical climate and equatorially trapped waves are generally well simulated in the superparameterized AMIP‐Style Simulation (Khairoutdinov et al., 2008). The role of the interhemispheric migration of the SST maximum in determining the spatial and temporal characteristics of TISO is evaluated with the help of following experiments:

- Sp4f19_10_10: This is the control run for boreal winter. The model is initialized on the 1st of October and a simulation through the 30th of April (7 months) is produced, during which the model is forced with the SpCCSM4 monthly climatological SST for the same period (the 1st of October to the 30th of April). The experiment designation is MI MB, where MI is the initial condition month and MB is the initial month of the imposed boundary conditions. The insolation follows its mean annual cycle starting with month MI, while the boundary conditions evolve according to the annual cycle that begins with MB. In this case, the model is initialized on the 1st of October, So the solar zenith angle runs through its cycle for October to April. The boundary conditions are for the period October to April as well (MB = MI in this case).
- Sp4f19_10_04: The model is initialized on the 1st of October (MI = 10) and run through the 30th of April, while the boundary conditions are for the period the 1st of April to the 31st of October (MB = 04). This experiment tests the sensitivity to opposite season SST for boreal winter.
- Sp4f19_04_04: This is the control run for boreal summer. The model is initialized on the 1st of April (MI = 4) and run through the 31st of October, while the boundary conditions are for the period the 1st of April to the 30th of October (MB = 04).
- Sp4f19 04 10: The model is initialized on the 1st of April (MI = 04) and run through the 31st of October, while the boundary conditions are for the period the 1st of October to the 30th of April (MB = 10). This experiment tests the sensitivity to opposite season SST for boreal summer.

Each set of simulations is performed for 30 seasons.

**Figure 13:**
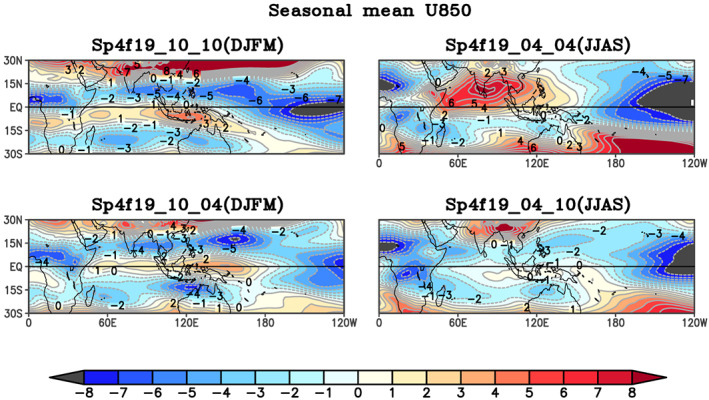
Seasonal mean low level zonal wind at 850 hPa in (a) Sp4f19_10_10, (b) Sp4f19_04_04, (c) Sp4f19_10_04, (d) Sp4f19_04_10 simulations. Units: m/s.

**Figure 14:**
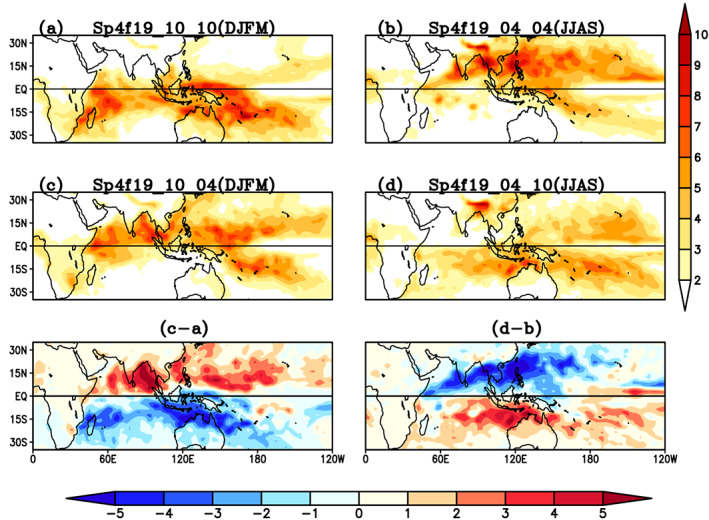
Lanczos band‐pass filtered standard deviation (20–100 days) of precipitation in (a) Sp4f19_10_10, (b) Sp4f19_04_04, (c) Sp4f19_10_04, (d) Sp4f19_04_10 simulations. Units: (mm/day).

The seasonal mean lower level zonal wind (850 hPa) from the experiments is shown in Figure 13. In the boreal winter control simulation, when SST and atmosphere are initialized in phase then low‐level westerly zonal wind is found between 5°N and 15°S (Figure 13a), while low‐level westerly zonal wind moves northward and found symmetric around the equator between 10°N and 10°S and maxima of low‐level zona wind is shifted eastward and located over the west Pacific Ocean when SST and atmosphere are initialized out of phase in Sp4f19_10_04 simulations. A stronger monsoonal circulation over the Indian subcontinent occurs when the SST and atmosphere are initialized in phase for boreal summer in control simulations (Sp4f19_04_04). In the Sp4f19_04_10 case, when the SST is specified for boreal winter and atmosphere is initialized for boreal summer, the low‐level westerly zonal wind is shifted toward the warm core of SST and reach south of the equator. Both sensitivity experiments when SST and atmosphere are initialized in different hemispheres show that low level circulation moves toward the warm SST hemisphere. Therefore, the position of low‐level westerly zonal wind is sensitive to the location of the warm core of the SST. The seasonal Lanczos band‐pass filtered standard deviation (20–100 day) of precipitation is shown in Figure 14. The intraseasonal variability in the control simulation (Figures 14a and 14b) can be seen to shift between the northern and southern hemispheres when SST and low level zonal circulation are in the boreal summer or boreal winter hemisphere, respectively. When SST and atmosphere are initialized out of phase, the intraseasonal variability shifts toward the warm core of SST (Figures 14c and 14d). The difference in intraseasonal variability between the in‐phase and out‐of‐phase simulations is shown in Figures 17e and 17f. The intraseasonal variability is increased in the hemisphere in which the SST maximum occurs. As we have seen in numerical experiments, interhemispheric migration of SST is a main driver of the seasonality of TISO.

**Figure 15:**
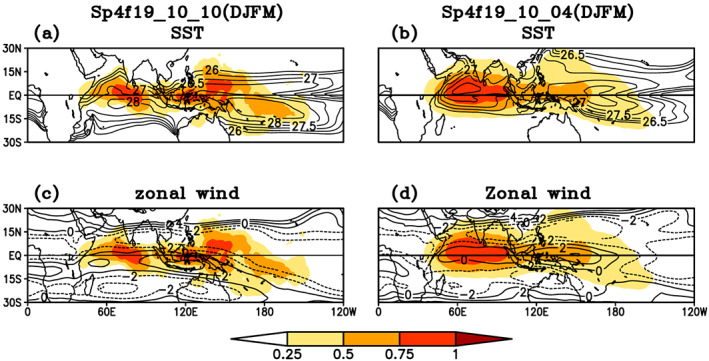
Composite of mean background states (contours) (a) sea surface temperature, (c) zonal wind (850 hPa), and TISO events tracks density (shaded) for Sp4f19_10_10 simulations; (b) sea surface temperature, (d) zonal wind, and TISO events tracks density (shaded) for Sp4f19_10_04 simulations.

**Figure 16:**
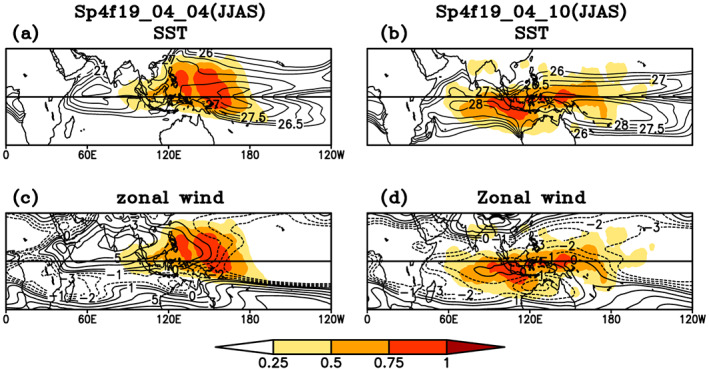
Composite of mean background states (contours) (a) sea surface temperature, (c) zonal wind (850 hPa), and TISO events tracks density (shaded) for Sp4f19_04_10 simulations; (b) sea surface temperature, (d) zonal wind, and TISO events tracks density (shaded) for Sp4f19_04_10 simulations.

To understand the occurrences of TISO, SST, and low level westerly zonal wind we have examine the coupling between the warm core of SST, the low‐level circulation, and track density index. We have used track density index instead of track density because number of tracks in each simulation are different and track density index is calculated by normalizing track density by maximum of track density in each simulation. The MOT algorithm was applied separately to each season for each experiment from the 16th of November to the 15th of April for boreal winter simulations and from the 16th of May to the 15th of October boreal summer simulations. Track density is calculated by using similar method as in observational analysis. Composite of SST low level westerly zonal wind and track density index in DJFM season is shown in Figure 15. The track density index for each simulation includes both northward‐ and eastward‐propagating TISO tracks. The track density for Sp4f19_10_10 simulations is a maximum where the warm core of SST and maximum zonal wind (≥−2 m/s) are collocated. Because convection in the model can occur for a relatively low threshold of SST, as compared to nature, and the low‐level circulation is weak in the tropics, the model has a relatively large spatial domain that is conducive for TISO as compared to observations (not shown). In Sp4f19_10_04 simulations, when SST is specified in boreal summer hemisphere, while atmosphere is initialized in boreal winter hemisphere and maximum low‐level zonal wind is shifted eastward and located over the west Pacific Ocean (Figure 15b), the track density for Sp4f19 10 04 simulations is shifted northward and maxima is now located over region, where the warm core of SST and maximum zonal wind (≥−2 m/s) are collocated. The maxima of the track density index are found over the warm core of SST and westerly wind in low‐level circulation as observed (Figures 15b and 15d). The coupling between the warm core of SST, low‐level circulation, and track density index in JJAS is shown in Figure 16. Similar to Figure 15, the maxima track density index in JJAS for Sp4f19_04_04 simulations are found in northern hemisphere. The track density index maximum is colocated with the warm core of SST and the weak low‐level circulation. TISO activity is less over Indian Ocean, even when the SST is warm and the zonal wind is strong westerly.

When SST is specified out of phase in JJAS (Sp4f19_04_10) case, the location of TISO activity is shifted to the southern hemisphere, but TISO activity is still found within the contours of warm SST and zonal wind (≥−2 m/s). From the observation analysis (Figure 12) and the sensitivity experiments (Figures 15 and 16), we have seen that the regionality and seasonality of the TISO events are closely coupled to the SST and low‐level circulation maxima. The geographical location of TISO events is controlled by the interhemispheric migration of the mean background SST and the low‐level circulation, which tends to follow the SST.

## 6 Conclusions

A new tracking method based on a MOT algorithm is developed to identify and track tropical convective anomalies from 20–100 days band‐pass filtered daily NOAA OLR anomalies from 1979–2017. Thresholds of size, intensity, and life span are used to detect events. Events are classified by their direction of propagation. The statistics of events obtained in this way are also comparable to those obtained in earlier studies using both subjective (Wang & Rui, 1990) and objective (Jones, Waliser, et al., 2004) methods. The manual identification in two‐dimensional space and time is tedious and can be difficult to reproduce, while the MOT algorithm can be applied to observational and simulated data sets and provides a richer set of metrics for multimodel comparison of TISO simulations. One advantage of MOT algorithm over other existing tracking methods is that it can objectively identifies if any tracks end prematurely and emerge later as a separate track, and joins these tracks as single track; therefore, it produces fewer number of tracks that dies over Maritime Continent. The algorithm also handles cases of splitting and merging of convective clusters. Verification of MOT algorithm identified TISO events with respect to well‐observed TISO events show that the MOT algorithm also reasonably captures other characteristics of TISO events including intensity, initiation, location, and dissipation location.

The indices, based upon the latitudinal average (RMM, ROMI, etc.), do not necessarily represent boreal summer MJO (Lee et al., 2013). The main advantage of the MOT algorithm is that it can track both eastward and northward propagating TISO events seamlessly without any assumption about season and without any latitudinal or longitudinal averages. RMM is also susceptible to influence by convectively coupled Kelvin waves (Roundy & Schreck, 2009), as occurred in the MJO‐2 event during the DYNAMO experiment when the RMM index amplitude was greater than one until phase 6. The MOT algorithm uses the 20–100 days band‐pass filtered signal. After filtering out high frequencies, the low frequency data and a smaller search radius enable this method to be less influenced by other convectively coupled modes. Both ROMI and the MOT algorithm use similarly filtered same data for signal identification; therefore, they more often agree. The MOT algorithm has the advantage over ROMI that it can track events in two‐dimensional space, while ROMI cannot. The MOT is a feature tracking method, which makes it very useful to apply to identify and track other propagating signals in time and space such as atmospheric rivers, monsoon low pressure systems, and tropical cyclones. There are some limitations to the MOT algorithm: the algorithm is designed to track the large convective clusters (minimum OLRA) related to the active phase of the convection, but TISO events are characterized by a dipole structure that includes both the active and suppressed phases. Also, MOT cannot be applied in real time because prefiltering of the signal is required.

In the analysis shown here, eastward‐moving tracks are found primarily south of the equator between 5°N and 15°S, initiating in the western to central Indian Ocean. After crossing the Maritime Continent, eastward‐moving TISO events turn southeastward near the South Pacific Convergence Zone. Northward propagation occurs in both the Indian and west Pacific Ocean basins. Two types of northward propagation happen in the Indian Ocean sector: (a) convective clusters that move northward immediately after initiation and (b) convective clusters that move eastward at first, then turn northward. TISO events originating in the Pacific Ocean sector propagate northwestward only. After initiation, northward propagating TISO events die north of 20°N. Both the eastward‐ and northward‐propagating components of TISO exhibit seasonality: eastward‐moving TISO events occur more often in boreal winter (DJFM), but they can occur throughout the year, while northward propagation is more common in boreal summer (MJJASO), although there are a few events in November and December. Because TISO events are found outside of the conventionally defined seasons, the conventional definition may underestimate TISO variability.

In the tropics, convection occurs where the troposphere is warm and moist. As the mean background state goes through its annual cycle and maxima move away from the equator slowly and continuously, the dominance of eastward propagation diminishes and the northward propagation becomes more prevalent. The seasonality of TISO in terms of the geographical location of occurrences and direction of propagation is primarily associated with the annual march of the maximum SST and low level zonal wind. When the low‐level circulation and the warm core of SST are straddling the equator, the primary direction of propagation is eastward. When the low level circulation and warm core of SST move into Northern Hemisphere, the primary direction of propagation is northward. Thus, the seasonality of TISO is tightly coupled with the mean background states of SST, moisture, and lower tropospheric circulation. TISO events, which are characterized by propagating deep convection, thrive where SST is warm, the atmosphere is moist, and the low‐level wind is westerly or weak easterly. As these characteristics of mean background state migrate between hemispheres, TISO events occur with different directions of propagation. The colocation of maxima of SST, moisture, and low‐level circulation, which is also known as Intratropical Convergence Zone, is a necessary condition for the regionality and seasonality of TISO. To address the relative roles of SST and low level circulation in the seasonality of TISO, a numerical modeling study in which SST and circulation is separated by a season shows that the seasonality of TISO in terms of the geographical location of occurrences and direction of propagation is primarily associated with the annual march of the maximum SST and low level zonal wind which tends to follow the SST. From the observation analysis and the sensitivity experiments, we have seen that the regionality and seasonality of the TISO events are closely coupled to the SST and low‐level circulation maxima.

## Supporting information

Supporting Information S1
