# Supplementary material for: Tracking of Tropical Intraseasonal Convective Anomalies: 1. Seasonality of the Tropical Intraseasonal Oscillations
Source: J Geophys Res Atmos. 2020 Feb 3;125(3):e2019JD030873. doi: 10.1029/2019JD030873 (PMC7422697; doi:10.1029/2019JD030873)
Supplement: Supplementary file 1 — Supporting Information S1 [file JGRD-125-e2019JD030873-s001.docx]

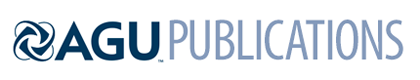


*Journal of Geophysical Research-Atmosphere*

Supporting information for

Tracking of Tropical Intraseasonal Convective Anomalies: Part1: Seasonality of the Tropical Intraseasonal Oscillations

Bohar Singh^1^, James L. Kinter^2^

^1^Colorado State University, Fort Collins, CO

^2^Center for Ocean-Land-Atmosphere Studies, George Mason University, Fairfax, VA

**Content of this file**

**Text S1:** Sensitivity of MOT algorithm to subjective parameters

**Text S2:** Data availability

**Figure:** S1

**Table:** S1-S3

**Introduction**

Text S1 show a sensitivity of subjective parameters to tracking of TISO. In Text 2 we provide the location of data repositories that used in this study. Figure S1 show sensitivity of MOT algorithm to various choice of subjective parameter that used in the algorithm. Tables S1-S3 show sensitivity of subjective parameters to initiation and dissipation dates.

Sensitivity of MOT algorithm to subjective parameters

**Text S1:** **MOT tracking method has three subjective parameters:**

(a) Search radius threshold parameter: This parameter is to draw an imaginary circle around predicted centroid of Convective Cloud System (CCS). Measured centroid found within this circle are considered as potential connection to update centroid position, if no measured CCS centroid found within this circle then event is declared dead and respective track is closed. Radius of the circle is controlled by search radius parameter. Sensitivity of a TISO track to search radius parameter threshold that occurred during DYNAMO field campaign between February 2012- April 2012 is shown in figure (S1(a)). As we can see that track of TISO events are not sensitive to the choice of search radius parameter ranging from 5-12 grid points. Number of grid points for search radius are chosen here on the basis of 2.5°×2.5° grid resolution of the data, it can change accordingly to the resolution of data, however TISO tracks are become sensitive search radius at values smaller than 5 grid points. Sensitivity of initiation and dissipation date are shown in table (S1) and we can see that initiation and dissipation date are also not affected by the choice of search radius parameter excepts for smaller values. Search radius threshold for the algorithm is chosen 6 grid points (15°).

(b) Object size threshold: Object size threshold is controlled the size of identified object during image analysis step. In MOT algorithm, object size threshold is kept 15 grid points (∼10^5^ km^2^), which is a well observed characteristic of TISO. Sensitivity of TISO tracking to object size threshold parameter is shown in figure(S1(b)) for search radius 6 grids and intensity threshold of -15 W/m^2^, as we can see that choice of size threshold does not affect the track location during mature stage of respective TISO event. However, initiation and dissipation are affected by 1-2 days (Table.S2).

(c) Intensity threshold is another well observed characteristic of the TISO. Other tracking method such as; WR90 and J04 used -15 w/m^2^ for intensity threshold, MOT algorithm is also used similar value of intensity threshold. Sensitivity of respective TISO event tracks to the choice of intensity threshold parameter is shown in figure (1S(c)). Respective TISO track is sensitive to intensity threshold parameter only during initiation and dissipation of the event, during mature state event is less sensitive to the choice of intensity threshold. As we can see from table (S3), initiation and dissipation date of the event are change by a week and location of initiation can change by 10°-15°. by the different choices of intensity threshold

MOT tracking algorithm is less sensitive to the choice of subjective parameter during mature stage of propagation. MOT algorithm is less sensitive to the choice of search radius and object size threshold in terms of initiation, dissipation date and location. While initiation, dissipation date and location are sensitive to the choice of intensity threshold by 2-7 days and 10°-15°respectively.

**Text S2: Data availability**

ERA-Interim data (Dee et al., 2011) were obtained on 8 September 2016 and are 60 available from the ECMWF public data set portal (<https://apps.ecmwf.int/datasets/data/interim-full-daily/levtype=sfc/)>

To identify and track TISO events, 39 years (1979-2017) of Outgoing Longwave Radiation (OLR; Liebmann & Smith, 1996); <https://www.esrl.noaa.gov/psd/data/gridded/data.interp_OLR.html)>

OISST v2 weekly data (Reynolds et al., 2007) is obtained from (<https://www.esrl.noaa.gov/psd/data/gridded/data.noaa.oisst.v2.highres.html)>

**Figure:** S1

**Figure S1**. Sensitivity of TISO event to (a) search radius threshold criteria (No. of grids) for Object size =15 grid points and intensity threshold =-15 (W/m^2^), (b) Object size threshold (No. of grids) for search radius = 6 grid points and intensity threshold =-15 (w/m2), (c) Intensity threshold criteria (w/m2) for Object size = 15 grid points and search radius 6 grid points

**Figure:** S1-S3

Table S1. Sensitivity of starting and end date of TISO to search radius (Dc) threshold criteria (No. of grids points)

| **SR. No.** | **Dc criteria** | **Starting date** | **End date** |
| --- | --- | --- | --- |
| 1 | 4 | 2012-03-01 | 2012-03-30 |
| 2 | 5 | 2012-02-13 | 2012-04-15 |
| 3 | 6 | 2012-02-13 | 2012-04-15 |
| 4 | 7 | 2012-02-13 | 2012-04-15 |
| 5 | 8 | 2012-02-13 | 2012-04-15 |
| 6 | 9 | 2012-02-13 | 2012-04-15 |
| 7 | 10 | 2012-02-13 | 2012-04-15 |
| 8 | 11 | 2012-02-13 | 2012-04-15 |
| 9 | 12 | 2012-02-13 | 2012-04-15 |

Table S2. Sensitivity of starting and end date of TISO to object size (No. of grid points) threshold criteria

| **SR. No.** | **Object size threshold** | **Starting date** | **End date** |
| --- | --- | --- | --- |
| 1 | 15 | 2012-03-11 | 2012-04-15 |
| 2 | 20 | 2012-02-13 | 2012-04-15 |
| 3 | 25 | 2012-02-13 | 2012-04-15 |
| 4 | 30 | 2012-02-14 | 2012-04-15 |

Table S3. Sensitivity of starting and end date of TISO to intensity threshold criteria (W/m^2^)

| **SR. No.** | **Intensity threshold** | **Starting date** | **End date** |
| --- | --- | --- | --- |
| 1 | -20 | 2012-03-03 | 2012-03-07 |
| 2 | -15 | 2012-02-13 | 2012-04-15 |
| 3 | -10 | 2012-02-10 | 2012-04-15 |
